# Alcohol Misuse and Kidney Injury: Epidemiological Evidence and Potential Mechanisms

**DOI:** 10.35946/arcr.v38.2.10

**Published:** 2017

**Authors:** Zoltan V. Varga, Csaba Matyas, Janos Paloczi, Pal Pacher

**Affiliations:** Zoltan V. Varga, M.D., Ph.D., is a visiting Research Fellow; Csaba Matyas, M.D., is a visiting Research Fellow; Janos Paloczi, Ph.D., is a visiting Research Fellow; and Pal Pacher, M.D., Ph.D., is a Senior Investigator and Lab Chief, all in the Laboratory of Cardiovascular Physiology and Tissue Injury, National Institutes of Health, National Institute on Alcohol Abuse and Alcoholism, Bethesda, Maryland

**Keywords:** Alcoholic nephropathy, nephrotoxicity, acetaldehyde, proteinuria, glomerular filtration rate (GFR), glomerulonephritis, alcohol use disorder (AUD), kidney injury

## Abstract

Chronic alcohol consumption is a well-known risk factor for tissue injury. The link between alcohol use disorder (AUD) and kidney injury is intriguing but controversial, and the molecular mechanisms by which alcohol may damage the kidneys are poorly understood. Epidemiological studies attempting to link AUD and kidney disease are, to date, inconclusive, and there is little experimental evidence directly linking alcohol consumption to kidney injury. However, studies conducted primarily in other organs and tissues suggest several possible mechanisms by which alcohol may promote kidney dysfunction. One possible mechanism is oxidative stress resulting from increased production of reactive oxygen species, which leads to an excessive amount of free radicals, which in turn trigger tissue injury and increase inflammation. In addition, AUD’s effect on other major organs (liver, heart, intestines, and skeletal muscle) appears to promote unfavorable pathological processes that are harmful to the kidneys. Notably, these mechanisms have not yet been validated experimentally in the kidney. Additional research is needed to clarify if alcohol does indeed promote kidney injury and the mechanisms by which alcohol-induced kidney injury may occur.

Alcohol use disorder (AUD) is a substantial public health problem, affecting 15.7 million people age 12 and older in the United States (Center for Behavioral Health Statistics and Quality 2016). In 2012, 5.9 percent of all global deaths were attributable to alcohol—7.6 percent for men and 4.0 percent for women. Moreover, alcohol-attributable deaths have increased worldwide, making alcohol the fifth leading risk factor for premature death and disability in 2010 and the first among people ages 15 to 49 (World Health Organization 2014).

Among the major consequences of chronic AUD that contribute to alcohol-related morbidity and mortality are liver cirrhosis, liver cancer, pancreatitis, and cardiovascular complications. To date, the epidemiological evidence connecting AUD and an increased incidence of chronic kidney disease is controversial. However, several preclinical studies suggest that alcohol consumption has a profound effect on the kidney and imply that there may be an independent pathologic entity, which we refer to here as “alcoholic kidney injury.”

Studies conducted primarily in other organs and tissues suggest several possible mechanisms by which alcohol may promote kidney dysfunction. In particular, alcoholic kidney injury may be associated with a complex interaction of ethanol-induced oxidative stress and pro-inflammatory alterations. This may be complicated by the interplay between the kidneys and other organs, including the liver, intestines, skeletal muscle, and cardiovascular system. This brief synopsis reviews the evidence in support of these hypotheses.

## Kidney Diseases and AUD: Lessons From Epidemiology

It is well established that cardiovascular diseases (including hypertension and ischemic heart disease) and diabetic microvascular complications are major risk factors for the development of chronic kidney diseases (Briasoulis et al. 2012; Carlsson et al. 2005; Reynolds et al. 2003; Ronksley et al. 2011). In turn, heavy alcohol consumption is implicated in the development of these cardiac diseases, with chronic, heavy drinkers at higher risk than those who consume small to moderate amounts of alcohol.

That said, epidemiological data have yet to confirm a relationship between alcohol consumption and chronic kidney disease. A recent meta-analysis (Cheungpasitporn et al. 2015) found little support for such a relationship. The researchers performed an extensive literature search using online databases (MEDLINE, EMBASE and Cochrane Databases) to identify studies investigating the association between high alcohol consumption and chronic kidney disease, end-stage renal disease, or proteinuria (i.e., excess protein in the urine that indicates kidney damage). Their analysis included 20 studies representing a total of 292,431 patients. The researchers reported that the pooled risk ratios of chronic kidney disease, proteinuria, and end-stage renal disease in patients with high alcohol consumption were 0.83, 0.85, and 1.00, respectively, indicating decreased risk or no risk of kidney disease in heavy alcohol consumers (Cheungpasitporn et al. 2015).

Other studies report similar findings, showing that the incidence of kidney disease is comparable or even lower in heavier drinkers (more than 210 g/week alcohol consumption) than in those who drink moderately (70–210 g/week alcohol consumption) (Buja et al. 2011; Knight et al. 2003; Koning et al. 2015; Reynolds et al. 2008; Sato et al. 2014; Yamagata et al. 2007). In contrast, some studies find that heavy alcohol consumption may predict poorer outcome in patients with chronic kidney diseases (Kronborg et al. 2008; Shankar et al. 2006; White et al. 2009). For example, White and colleagues (2009) reported that heavier drinkers (those consuming more than 30 g of alcohol/week) were at higher risk of incident albuminuria, which is typically a symptom of kidney disease. Japanese (Yamagata et al. 2007) and Italian (Buja et al. 2011) cohort studies revealed a U-shaped association between alcohol consumption and incidence of proteinuria. It is possible that the contradictory findings are the result of varying effects of different types of alcoholic beverages on the kidney, or the result of different alcohol consumption patterns in different countries. In addition, the self-reporting nature of drinking behaviors and the amount of alcohol consumed may bias some of the conclusions as shown, for example, by Parekh and Klag (2001), who found that people who drink heavily underreport their alcohol consumption.

## Potential Mechanisms of Alcoholic Kidney Injury: Lessons From Experimental Studies

If alcohol consumption does in fact influence kidney disease, the question remains: How? There is direct and indirect evidence for several possible mechanisms. These changes are caused either by alcohol itself or by excessive amounts of the products formed when cells break down (or metabolize) alcohol, including acetaldehyde, NADH, and free radicals. These alcohol-related pathophysiologic changes in cells have been linked to damage in many organs and may play a role in kidney damage. In addition, complex interactions between organs may further complicate and accentuate the development of kidney pathology in people with AUD (see figure).

### Oxidative Stress

Free radicals (also called reactive oxygen species [ROS]) are one of the by-products of alcohol metabolism and are known to cause cellular damage, unless the body can use antioxidants to clean them up. Oxidative stress occurs when the body cannot detoxify free radicals as fast as they are being produced, and it is pivotal in triggering alcohol-related tissue injury. Studies suggest that several mechanisms produce ROS in alcohol-damaged organs, including the liver (Cederbaum et al. 2009), heart (Tan et al. 2012; Varga et al. 2015), and kidney (Latchoumycandane et al. 2015). The mechanisms producing ROS in organs include nonenzymatic mechanisms such as mitochondrial electron transport chain malfunction (Gyamfi et al. 2012; Mantena et al. 2008) and enzymatic mechanisms that involve enzymes such as NADPH oxidases (Kono et al. 2000) and the enzyme CYP2E1 (Lu and Cederbaum 2008). CYP2E1 is of particular interest when thinking about potential mechanisms for alcohol-related kidney damage. The body mainly metabolizes alcohol using the enzyme alcohol dehydrogenase, which is expressed primarily in the liver. However, during chronic ethanol consumption, the body also uses CYP2E1 in the liver as well as the kidneys. Interestingly, studies find that CYP2E1 induction is much more robust in the kidneys compared with the liver (Roberts et al. 1994; Zerilli et al. 1995). This massive induction of CYP2E1 in the kidneys results in oxidative stress that modifies phospholipids in cell membranes. Such modified phospholipids may in turn activate immune cells called neutrophil granulocytes, which further aggravates oxidative stress, promoting a vicious cycle (Latchoumycandane et al. 2015).

Studies suggest that ethanol consumption may increase renal expression of other potential sources of free radicals involving a family of enzymes called nitric oxide synthases (Tirapelli et al. 2012). Nitric oxide synthase stimulates the production of nitric oxide, which, if produced excessively, can react with other molecules and create free radicals that trigger tissue damage in the kidneys (Pacher et al. 2007; Szalay et al. 2015). Tirapelli and colleagues (2012) showed that ethanol consumption increased the expression of two nitric oxide synthases. However, it is still unclear exactly how ethanol upregulates nitric oxide synthases, or whether it does so directly or indirectly. It may be that toxins released from the intestines into blood circulation because of ethanol’s effects on the digestive system activate the expression of nitric oxide synthase. Another theory suggests that both enzymes may undergo the process of uncoupling due to oxidation or lack of critical coenzymes (e.g., tetrahydrobiopterin). Uncoupling eventually leads to generation of damaging ROS like superoxide anion, instead of the vasorelaxant nitric oxide that maintains normal blood flow in the kidney.

### Alcohol-Metabolism Derived Intermediaries

Along with oxidative stress, increasing evidence suggests that some nonoxidative mechanisms also factor into alcohol-related organ damage. Specifically, ethanol metabolism produces fatty acid ethyl esters in various organs (Laposata and Lange 1986), which can cause ethanol-induced organ damage. Calabrese and Rizza (1999) found that ethanol induced a significant increase in the levels of fatty acid ethyl esters. They measured the highest levels in the heart, followed by kidney, brain, and liver.

Due to the metabolism of ethanol, significant amounts of acetate are produced and subsequently incorporated into acetyl-coenzyme-A, a molecule that participates in metabolism of proteins, lipids, and carbohydrates. This leads to the reprogramming of systemic metabolism. Protein acetylation—adding an acetyl group to a protein—is integral to regulating processes controlled by mitochondria, including fatty acid metabolism and antioxidant defense (Choudhary et al. 2014). Our current understanding is that the balance of lysine acetylation and deacetylation (the removal of an acetyl group) of key proteins (e.g., of the master regulator of mitochondrial biogenesis, PGC-1 alpha) serves, at least in part, to trigger a switch in metabolic status in conditions of overnutrition or undernutrition (Bai et al. 2015; Ghanta et al. 2013; Jeninga et al. 2010). A recent study demonstrated that ethanol induces mitochondrial protein hyperacetylation (excessive modification by acetylation of the lysine residues of a protein) in the kidney, which might interfere with the function of some mitochondrial proteins involved in alcohol metabolism or defense against oxidative stress (e.g., superoxide dismutase 2, aldehyde dehydrogenase 2, gluthatione peroxidase). This could also be a significant factor contributing to ethanol-induced mitochondrial dysfunction in the kidneys (Harris et al. 2015).

### Alcohol-Induced Intestinal Damage

Alcohol-induced intestinal damage and increased mucosal translocation of bacterial endotoxin are crucial in the initiation and progression of alcoholic liver injury and in the pathogenesis of other alcohol-related diseases (Bala et al. 2014; Purohit et al. 2008). (For an in-depth discussion of alcohol and the digestive tract, see the article by Keshavarzian in this issue.) The direct role of alcohol-related endotoxin release in alcoholic kidney injury has not yet been studied. However, it is possible that activation of the innate immune system due to endotoxins released by a leaky gut plays a central role in the development of renal damage, as it does for liver damage (Zhang et al. 2008).

Substantial experimental and clinical evidence suggests that increased intestinal permeability and endotoxin release caused by excessive alcohol consumption leads to higher levels of circulating immunoglobulin A (IgA), an antibody critical to the immune response of mucous membranes. The kidney is particularly sensitive to an increased IgA load. In fact, IgA glomerulonephritis—acute inflammation of the kidney caused by an IgA immune response—is one of the most common types of primary glomerulonephritis worldwide (D’Amico 1987). This IgA-related kidney disease leads to clinical symptoms of renal injury and eventually progresses into renal failure (Amore et al. 1994; Bene et al. 1988; Pouria and Feehally 1999). Experimental studies suggest that heavy alcohol consumption induces IgA kidney disease (Smith et al. 1990). In addition, rats given intragastric infusions of a commercial whiskey (1.5 ml/100 gm body weight) 3 times a week along with a nutrient-deficient diet develop a more severe form of IgA nephropathy (Amore et al. 1994).

Evidence also exists that alcohol-related damage to the liver, in particular advanced liver cirrhosis, leads to hepatorenal syndrome (HRS)—a deterioration in renal function related to impaired circulation. The underlying mechanisms involved in the development and progression of HRS are incompletely understood, although it is plausible that the altered balance between vasoconstrictor and vasodilator factors plays a significant role (Lenz 2005).

### Alcoholic Skeletal Myopathy: A Potential Indirect Mechanism

Severe AUD is frequently associated with various acute or chronic muscle symptoms, including difficulties with gait, muscle cramps, pain, and overall reduced muscle mass. In fact, biochemical lesions in the muscles and the resulting myopathy develop independently of any peripheral neuropathy, macro- and micronutrient malnutrition, and overt liver disease in people with AUD. In chronic alcoholic myopathy, a person’s entire muscle mass may be reduced by up to one-third. It is the most common skeletal muscle disorder in the industrialized world, present at varying severity in approximately half of alcohol misusers (Preedy et al. 2001). To date, studies have not examined whether there is a direct link between acute alcoholic myopathy and kidney injury. However, several lines of research suggest there might be a connection.

Although the mechanism of alcoholic myopathy is not fully understood, it is likely that disruption of mitochondria-related energy homeostasis is important in promoting muscle cell (myocyte) injury (Eisner et al. 2014). In rare cases in malnourished chronic alcoholics, acute alcoholic myopathy, also termed acute alcoholic necrotizing myopathy or alcoholic rhabdomyolysis, also may occur, which may lead to reversible or irreversible acute kidney injury (Haller and Knochel 1984; Hewitt and Winter 1995; Muthukumar et al. 1999; Sofat et al. 1999).

A few studies have linked rhabdomyolysis and myoglobin toxicity with acute kidney injury, supporting a possible association among alcohol use, alcohol-related acute myopathy, and kidney damage. For example, Belliere and colleagues (2015) showed a link between rhabdomyolysis and excessive macrophage infiltration in the kidney, which in turn led to pro-inflammatory marker expression and consequent tissue injury (Belliere et al. 2015). Another study by Plotnikov and colleagues (2009) showed that mitochondria isolated from rat kidneys were damaged by oxidative stress when incubated with myoglobin. This finding suggests that rhabdomyolysis and myoglobin toxicity may trigger oxidative stress in the kidney via mitochondrial injury.

### Alcoholic Cardiomyopathy: Another Potential Confounder

Several epidemiological studies have shown that mild alcohol consumption benefits cardiovascular health (Coate 1993; Kannel and Ellison 1996) by reducing the risk of coronary heart disease (Mukamal et al. 2006). In contrast, heavy drinking leads to the development of nonischemic dilated cardiomyopathy (Klatsky 2007) and significantly increases the risk of sudden cardiac death (Hookana et al. 2011).

Chronic or acute heart failure can lead to chronic or acute dysfunction in the kidneys, known as cardiorenal syndrome (Cleland et al. 2012). The complex renal pathophysiological response leads to fluid buildup in tissues, ischemic injury, peripheral vasoconstriction, and activation of the hormone system that helps regulate blood flow (called the renin–angiotensin–aldosterone system, or RAAS) (Palazzuoli and Ronco 2011). The overactivation of RAAS further aggravates oxidative stress in chronic alcoholism (Ungvari et al. 2004). As a consequence, oxidative stress not only propagates kidney failure, but it also contributes to the progression of chronic heart failure (Pacher et al. 2005) and leads to a vicious cycle in alcohol-induced cardiovascular complications.

## Conclusions

As noted above, there is much to learn about alcoholic kidney disease and the complex interplay among multiple organs affected by alcohol consumption. Although research suggests several potential mechanisms by which alcohol may directly or indirectly affect the kidneys, they have not yet been validated experimentally. Future research will hopefully explore these hypotheses to provide a better understanding of alcoholic kidney injury. This article highlights the effects of other organs on kidney and renal function; however, it should be noted that alcoholic kidney injury itself may have negative metabolic consequences. One such complication is impaired vitamin D metabolism (Shankar et al. 2008), which may influence the function of several other organs, creating a vicious cycle.

The treatment of alcoholic kidney injury is still largely symptomatic, despite accumulating knowledge about underlying mechanisms. Both preclinical and human studies highlight the central role of oxidative stress and inflammation in triggering and driving the pathological processes associated with alcoholic kidney injury. Early diagnosis of this condition and rigorous abstinence from alcohol are very important for slowing down the progression of the disease and allowing the kidneys to regenerate.

## Figures and Tables

**Figure:**
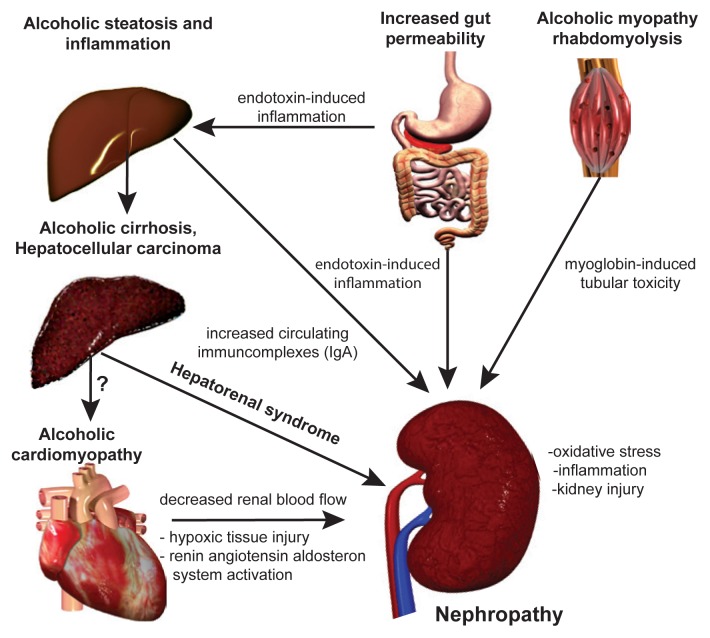
Possible mechanism for alcohol-induced kidney injury. Chronic alcohol consumption induces profound injury in several organs that may affect and aggravate the deleterious effect of ethanol on the kidney. Ethanol itself markedly induces the expression of the microsomal ethanol oxidation system (CYP2E1), producing reactive oxygen species as a byproduct. Increased gastrointestinal permeability and endotoxin load may lead to alcoholic steatohepatitis resulting in excessive immunoglobulin A (IgA) load (due to increased intestinal production and decreased hepatic IgA clearance). IgA deposits may accumulate in the kidney, leading to glomerulopathy. Renal microcirculatory alterations in advanced liver cirrhosis leads to hepatorenal syndrome. Alcohol-induced skeletal muscle damage leads to excessive amounts of circulating myoglobin, causing renal tubular injury as a result of increased oxidative stress. Due to the development of alcoholic cardiomyopathy, chronic renal hypoxia develops, activating the renin–angiotensin–aldosterone system (RAAS), which in turn leads to further free radical production and to the propagation of fibrotic pathways.
